# Supplementary material for: Genetic Diversity and Population Genetic Structure of Aedes albopictus in the Yangtze River Basin, China
Source: Genes (Basel). 2022 Oct 26;13(11):1950. doi: 10.3390/genes13111950 (PMC9690033; doi:10.3390/genes13111950)
Supplement: Supplementary file 1 [file genes-13-01950-s001.zip › genes-1875632-supply.pdf]

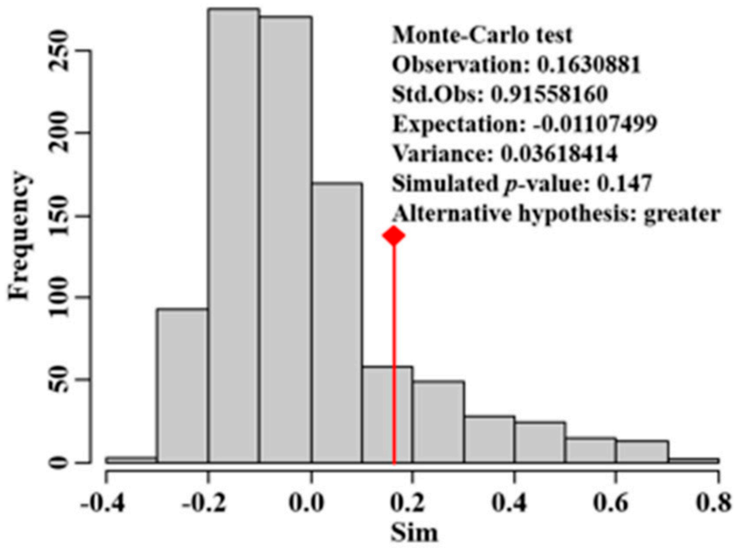

Supplemental figure S1. IBD analysis of Ae. albopictus population in the Yangtze River Basin.

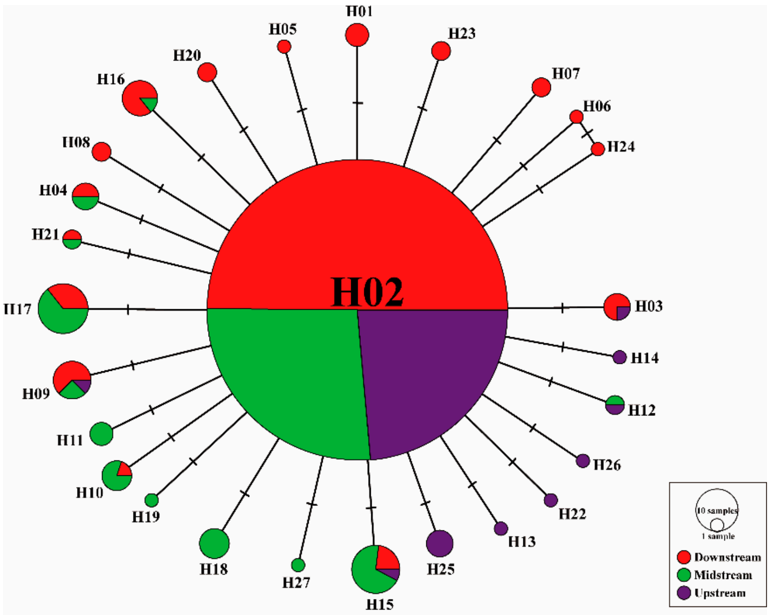

Supplemental figure S2. Haplotype network analysis of 22 Ae. albopictus populations in the Yangtze River Basin based on *CoxI* gene.

Supplemental table S1. Analysis of molecular variance (AMOVA) test of 22 *Ae. albopictus* populations sampled from 7 locations of the Yangtze River Basin.

Source of variation	d.f.	Sum of squares	Variance components	Percentage of variation	Fixation Indices
Among populations	6	81.4	0.06248 Va	2.03	0.0203 (p<0.00001)
Among individuals	579	2057.956	0.53826 Vb	17.48	0.17846 (p<0.00001)

within populations within individuals	586	1452	2.47782 Vc	80.49	0.19514 (p<0.00001)
Total	1171	3591.356	3.07855		
